# Revisiting Depression Rating Scales: Analysis of a Randomized Trial

**DOI:** 10.7759/cureus.71346

**Published:** 2024-10-13

**Authors:** N Simple Santi, Sashi B Biswal, Birendra Narayan Naik, Jyoti Prakash Sahoo, Bhabagrahi Rath

**Affiliations:** 1 Pharmacology, Veer Surendra Sai Institute of Medical Sciences and Research (VIMSAR), Burla, IND; 2 Psychiatry, Veer Surendra Sai Institute of Medical Sciences and Research (VIMSAR), Burla, IND; 3 Pharmacology, Kalinga Institute of Medical Sciences, Bhubaneswar, IND

**Keywords:** bias, bland-altman analysis, hamilton depression rating scale, limits of agreement, major depressive disorder, measurement error, montgomery-asberg depression rating scale, precision, randomized clinical trial, variance

## Abstract

Background and objectives: The Hamilton Depression Rating Scale (HDRS) and Montgomery-Åsberg Depression Rating Scale (MADRS) are both frequently employed to gauge the symptoms of major depressive disorder (MDD). Limited studies have attempted to compare these two scales. The purpose of our study was to compare the HDRS and MADRS scores of the study population as well as to calculate their bias and precision.

Methods: An open-label, randomized, three-arm trial was executed on 96 MDD patients. For 16 weeks, participants were put into three groups (in a 1:1:1 ratio) and administered vortioxetine (5-20 mg/day), escitalopram (10-20 mg/day), or vilazodone (20-40 mg/day). Vortioxetine and vilazodone were experimental drugs, while escitalopram acted as a control. Follow-up visits were scheduled at four-week intervals following the first visit. The per-protocol (PP) population had their HDRS and MADRS scores recorded at each visit. We contrasted the MADRS and HDRS scores through the Bland-Altman analysis and Taffé method. The former method generated the limit of agreement (LoA) plot. Moreover, the latter provided the bias, precision, and comparison plots. We prospectively registered our trial in the Clinical Trial Registry of India (CTRI) (2022/07/043808).

Results: We screened 134 individuals, and 109 (81.34%) were qualified. Ninety-six (88.07%) accomplished the 16-week study. The average age was 46.3 ± 6.2 years. The study population had baseline and final HDRS scores of 30.05 ± 1.52 and 14.29 ± 1.51, respectively. The mean MADRS scores at the first and last visits were 35.73 ± 1.47 and 18.08 ± 1.92, respectively. According to the LoA plot, the mean difference between HDRS and MADRS scores was 4.78 (95% CI: 2.61-6.95). As the bias plot indicates, MADRS scores had an estimated differential bias of 3.478 (95% CI: -2.119 to 9.074) and a proportional bias of 0.816 (95% CI: 0.649 to 0.982). The precision plot demonstrated that HDRS scores had lower standard deviation values and a narrower confidence interval than MADRS scores. This meant that the HDRS was more precise. The comparison plot showed that the regression line of the recalibrated MADRS coincided with that of HDRS. The MADRS was effectively recalibrated to remove bias.

Conclusion: After 16 weeks of therapy, we noticed substantial drops in HDRS and MADRS scores. The HDRS was proved to be more precise than the analogous MADRS. The MADRS was recalibrated to remove differential and proportional biases.

## Introduction

In the last two decades, the global prevalence of major depressive disorder (MDD) has intensified profoundly [1]. Hamilton Depression Rating Scale (HDRS) is the most extensively used measure for assessing depression symptoms to diagnose and track therapy [2]. Montgomery-Åsberg Depression Rating Scale (MADRS) is also commonly used to measure the effectiveness of antidepressant drugs [3]. HDRS and MADRS provide objective assessments of depression symptoms. Beck Depression Inventory (BDI) is a commonly acknowledged subjective assessment tool to diagnose and monitor depression [4]. Other commonly used scales are as follows: Hospital Anxiety and Depression Scale (HADS) [5], Geriatric Depression Scale [6], and Patient Health Questionnaire-9 (PHQ-9) [7]. The comparisons between HDRS and MADRS are attracting adequate attention. Furthermore, the precision of these ratings in diagnosing and tracking depression symptoms has not been estimated.

Based on our literature review, we considered conducting this trial with three antidepressants: vortioxetine, escitalopram, and vilazodone. We employed HDRS and MADRS to gauge the efficacy of these medications. Our study's interim and final assessments demonstrated the efficacy and safety of these medicines [8,9]. We also investigated the effects of these medications on quality of life, patient compliance, and metabolic markers [10-13]. The Bland-Altman analysis [14] is extensively used to determine the interchangeability of two quantitative scales. During our interim analysis, we utilized this method to compare MADRS with HDRS [15].

In clinical studies, both differential and proportional biases exist. These biases and measurement errors contribute to the disparity between observed and expected values [16]. The Bland and Altman's limits of agreement (LoA) plot [14] visually illustrates the agreement between the two methods. The LoA plot is constructed with the premise that both methods' precision and measurement error variances remain constant [16]. It also presupposes that there is only a differential bias [16]. The Taffé method addresses both differential and proportional biases. This method makes the analysis apparent and understandable with bias, precision, and comparison plots [16,17].

In this study, we analyzed the HDRS and MADRS scores of the study participants through Bland and Altman's limits of agreement (LoA) plot [14] and the Taffé method [17].

## Materials and methods

We evaluated the HDRS and MADRS scores of our study participants in this randomized, open-label trial. The study spanned from July 2022 to December 2023. The study site was a tertiary care government hospital (i.e., Veer Surendra Sai Institute of Medical Sciences and Research (VIMSAR), Burla) in India. We obtained ethical permission (029-2022/I-S-T/03) before the commencement of the trial. Each participant signed an informed consent form. We prospectively registered for our study at the Clinical Trial Registry of India (CTRI) (2022/07/043808). The study followed institutional norms, good clinical practices, and the Declaration of Helsinki.

Study criteria

We included adult patients (18-65 years) diagnosed with MDD and a baseline HDRS score of 24 or above. Exclusion criteria included any confirmed allergy to study drugs, severe renal impairment, organic brain disorders, psychotic symptoms, or a thromboembolic incident within the previous six months. Furthermore, this study excluded nursing mothers and pregnant women. The subjects were free to deny their participation whenever they felt so.

Study design and objectives

Vortioxetine and vilazodone were the experimental drugs, with escitalopram operating as the control. The enrolled participants were sorted into three groups to ensure randomization: A, B, and C. The allocation ratio was set at 1:1:1. Groups A, B, and C got tablets of vilazodone (20-40 mg), escitalopram (10-20 mg), and vortioxetine (5-20 mg), respectively. We used permuted block randomization with 12- and 24-size blocks. We stratified randomization by MDD duration and gender. The study objective was to compare the HDRS and MADRS scores of the participants using the Bland-Altman analysis [14] and the Taffé method [17]. The analyses centered on the per-protocol (PP) population.

Study procedure

Group A subjects got 20-40 mg of vilazodone tablets once daily. Similarly, B- and C-group subjects had 10-20 mg/day of escitalopram and 5-20 mg/day of vortioxetine, respectively. The psychiatrist tailored the dose following the patient's clinical presentation. The crossover of drugs was not authorized. The baseline visit assessed participants' psychological and physical health comprehensively. Following the first appointment, we slated follow-up visits to gauge HDRS and MADRS scores after 4, 8, 12, and 16 weeks from the baseline. The scores were examined using the Bland-Altman analysis [14] and the Taffé method [17]. We also used Pearson correlation to link the individual scores and changes from baseline.

Study tools and assessments

Bland-Altman Analysis

Bland and Altman's limit of agreement (LoA) plot is used to gauge the compatibility between two quantitative scales. In 1983, Altman and Bland proposed an analysis that quantifies agreement between two quantitative indicators by calculating the mean difference and defining the limits of agreement [18]. To assess method comparability, one must compile measurements from multiple methodologies for various subjects. The limits (i.e., LoA) are computed by subtracting and adding 1.96 times the standard deviation from the mean difference. The differences and means generate a scatter plot. The plot includes a regression of differences to determine bias and its direction. Data was computed using R version 4.4.1 (The R Foundation for Statistical Computing, Vienna, Austria) [19]. We used the BlandAltmanLeh package [20] to generate the LoA plot [14].

Taffé Method

Limits of agreement might be misleading when the variances of the measurement errors of both approaches differ significantly. In 2016, Taffé developed an R package, i.e., MethodCompare, to adopt a new statistical approach [21]. The MethodCompare package creates three plots to juxtapose the effectiveness of new and reference scales. These plots are regarded as bias, precision, and comparison plots [17,18].

In our study, we considered HDRS and MADRS as the reference standard and new methods, respectively. The Taffé technique computed the HDRS regression model with marginal maximum likelihood. It permitted the error variances for the mean of individual measurements. This serves as a rough approximation of projected values [18]. Using an empirical Bayes technique, values are predicted based on the mean of their posterior distribution, resulting in the best linear unbiased prediction (BLUP) for the value (denoted as “x”) [18]. Including a smooth estimation of heteroscedasticity independent of HDRS scores and instead depending on the BLUP of x is recommended. The Taffé method, similar to Bland and Altman, involves regressing the absolute error values of the linear regression model to obtain a smooth estimate of heterogeneous variance [18].

A scatter plot was obtained using the MADRS, HDRS scores, and BLUP of x. Two regression lines were drawn for each tool. An additional scale on the right displayed the correlation between anticipated values and bias [18]. This plot is known as the bias plot. The simulation demonstrated that the differential and proportional bias estimations are consistent and unbiased for the repeated MADRS and HDRS measurements. The recalibration technique reduced the MADRS's differential and proportional biases. The comparison plot illustrated the recalibrated values of MADRS and their comparison to the HDRS scores. We adopted the precision plot to compare the variances by plotting the estimated standard deviations and the BLUP of x. The MethodCompare package [21] generates bias, precision, and comparison plots based on the stated method.

Statistical analysis

With a mean HDRS reduction of 10.0 from baseline and a standard deviation (SD) of 2.0, 87 subjects (29 in each group) were demanded to measure an alteration in HDRS with 80% power and a 0.05 two-sided significance level. We finalized 96 cases to permit a 10% dropout rate. We pulled off an interim analysis once the first 48 patients' 12-week visits had been accomplished.

The Shapiro-Wilk test was adopted to validate the normality of data distribution. The mean with standard deviation (SD) was provided for continuous data. As a result, they were evaluated using analysis of variance (ANOVA). The categorical data were displayed in terms of frequency and proportion. Hence, Pearson's chi-square (ꭓ^2^) test was utilized for assessment. The correlation coefficients and 95% confidence intervals (CI) were presented. We leveraged R software version 4.4.1 [19]. A p-value of 0.05 or lower was deemed statistically significant.

## Results

**Figure 1:**
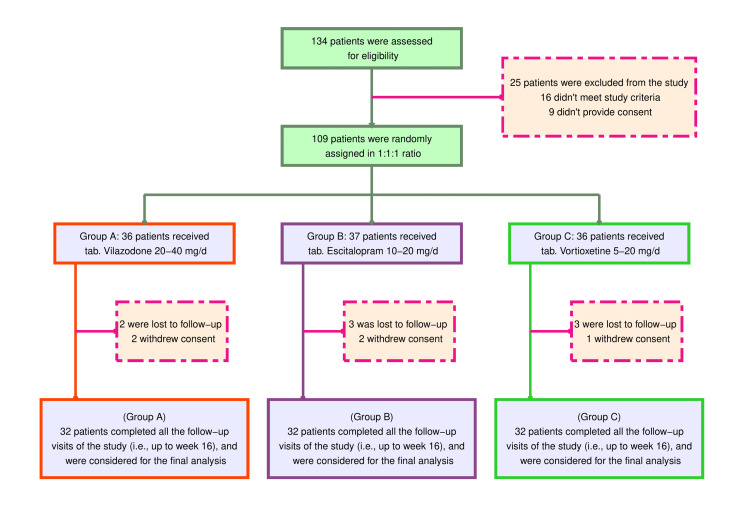
The CONSORT diagram CONSORT: consolidated standards of reporting trials

This randomized trial probed 134 MDD patients (Figure 1). Twenty-five individuals were scrubbed from the study. Sixteen people were designated ineligible, and nine refused to participate. Hence, 109 patients were randomly allocated to either of the three study groups. Of the recruited subjects, eight were lost to follow-up and five revoked their consent. The rest of the 96 subjects were evaluated. They had comparable baseline attributes across the three groups of study participants (Table 1).

**Table 1:** Baseline traits of the study population (n = 96) The category data were displayed using the n (%) format. The continuous variables were represented using the mean ± SD. BMI: body mass index; HDRS: Hamilton Depression Rating Scale (17-item version); MADRS: Montgomery Åsberg Depression Rating Scale; T/T naïve: a newly diagnosed or treatment-naïve patient

Parameter	Total (n = 96)	Group A vilazodone (n = 32)	Group B escitalopram (n = 32)	Group C vortioxetine (n = 32)	p-value
Age (years)	46.3 ± 6.2	47.1 ± 6.4	46.0 ± 5.5	45.7 ± 6.1	0.143
Age group					
≤50 years	64 (66.7%)	23 (71.9%)	20 (62.5%)	21 (65.6%)	0.580
>50 years	32 (33.3%)	9 (28.1%)	12 (37.5%)	11 (34.4%)	
Gender					
Female	48 (50.0%)	16 (50.0%)	16 (50.0%)	16 (50.0%)	1
Male	48 (50.0%)	16 (50.0%)	16 (50.0%)	16 (50.0%)	
Marital status					
Married	72 (75.0%)	25 (78.1%)	23 (71.9%)	24 (75.0%)	0.477
Unmarried	24 (25.0%)	7 (21.9%)	9 (28.1%)	8 (25.0%)	
Education level					
Literate	80 (83.3%)	27 (84.4%)	26 (81.2%)	27 (84.4%)	0.189
Illiterate	16 (16.7%)	5 (15.5%)	6 (18.8%)	5 (15.5%)	
Duration of disease					
T/t naïve	48 (50.0%)	16 (50.0%)	16 (50.0%)	16 (50.0%)	1
<6 months	48 (50.0%)	16 (50.0%)	16 (50.0%)	16 (50.0%)	
BMI (kg/m^2^)	27.3 ± 4.8	26.4 ± 4.1	27.7 ± 5.2	27.8 ± 4.5	0.028
HDRS	30.05 ± 1.52	30.06 ± 1.50	29.94 ± 1.34	30.16 ± 1.74	0.964
MADRS	35.73 ± 1.47	35.81 ± 1.60	35.81 ± 1.23	36.03 ± 1.67	0.741

**Figure 2:**
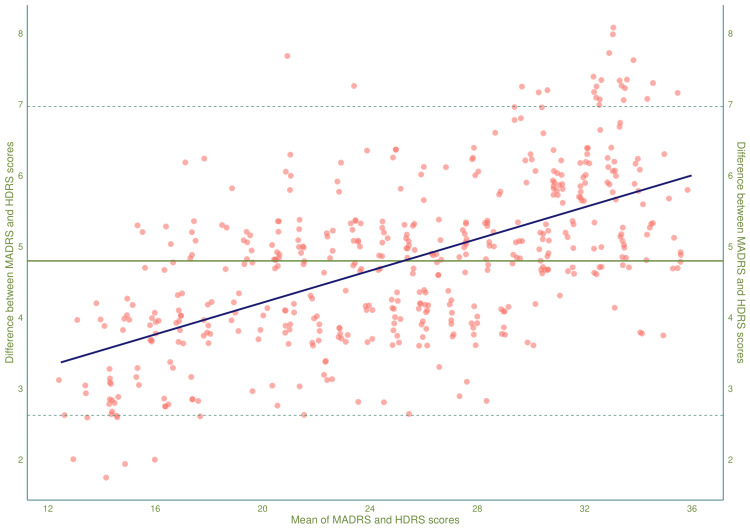
Bland-Altman’s limit of agreement (LoA) plot showing HDRS and MADRS scores of the study participants The Bland-Altman plot compares every research participant's HDRS and MADRS values during 16 weeks of study duration. The X- and Y-axes show the average and differences of the two scores. The mean difference and confidence intervals are shown through the solid and dotted horizontal lines. The blue line depicts the relationship between mean values and score differences. HDRS: Hamilton Depression Rating Scale-17 items version; MADRS: Montgomery-Åsberg Depression Rating Scale

Figure 2 showcases the Bland-Altman's limit of agreement (LoA) plot. It compares the means and differences of the study population's corresponding HDRS and MADRS scores during the entire study period. The LoA plot shows the upper and lower limits of the difference between the scores as horizontal dotted lines. The mean difference between the scores was 4.78 (95% CI: 2.61-6.95). With the advancement of study duration, the individual scores, along with the mean scores, reduce. The difference between the scores narrows down with the reduction of mean scores. It implies that the HDRS and MADRS scores can be utilized interchangeably for milder cases of depressive disorders. However, the LoA plot indicates positive and negative biases for the high and low scores.

**Figure 3:**
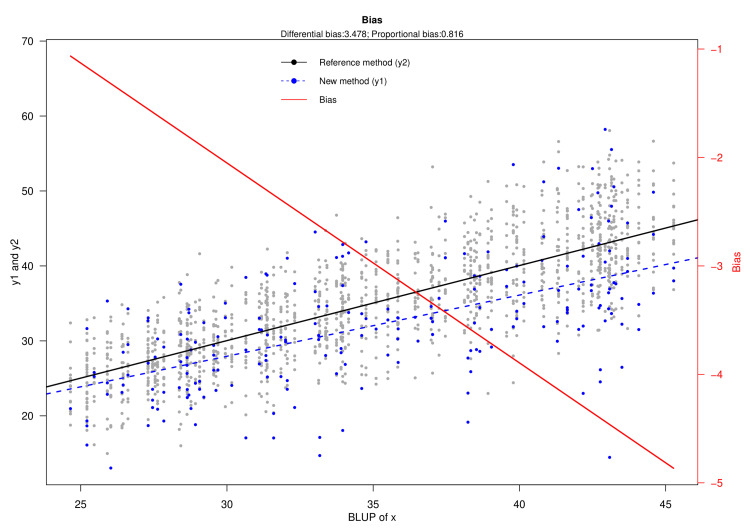
Bias plot The X-axis represents the BLUP of x. The Y-axis shows HDRS and MADRS scores. The new method (designated as y1) is shown in blue. Furthermore, the reference method (designated as y2) is shown in black. The blue and black lines represent the regression lines for the corresponding values. The red line illustrates the bias of the new method (i.e., MADRS). HDRS: Hamilton Depression Rating Scale; MADRS: Montgomery-Åsberg Depression Rating Scale; BLUP: best linear unbiased prediction

Figure 3 portrays the bias plot for the HDRS and MADRS scores. The X- and Y-axes represent the BLUP of x and the scores assessed with MADRS (shown as y1) and HDRS (shown as y2). The plot illustrates the bias of the new measurement method (i.e., MADRS) juxtaposed with the reference method (i.e., HDRS). The estimated differential and proportional biases are 3.478 (95% CI: -2.119 to 9.074) and 0.816 (95% CI: 0.649 to 0.982), respectively. The bias becomes more negative as the true latent trait (i.e., BLUP of x) increases. The scores of the two measurement methods are heteroscedastic. Moreover, this heteroscedasticity increases with the rising BLUP of x. Nonetheless, the dispersion of MADRS scores is greater than HDRS scores.

**Figure 4:**
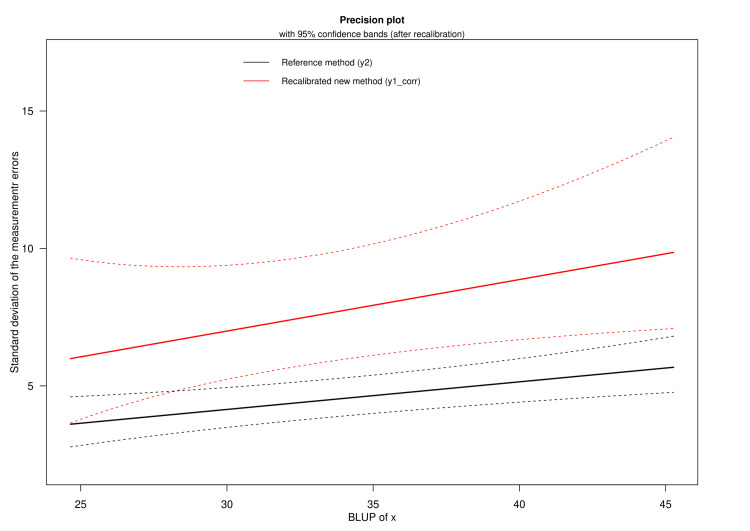
Precision plot The BLUP of x and the standard deviation of the measurement error are illustrated in the X- and Y-axes, respectively. The recalibrated new method (i.e., MADRS, designated as y1_corr) is shown in red. And, the reference method (i.e., HDRS, designated as y2) is black. The red and black lines represent the precision plot for the corresponding methods. The dotted lines imply the 95% confidence interval. HDRS: Hamilton Depression Rating Scale; MADRS: Montgomery-Åsberg Depression Rating Scale; BLUP: best linear unbiased prediction

Figure 4 displays the precision plot of the reference method (i.e., HDRS) and the recalibrated new method (i.e., MADRS). The X- and Y-axes represent the BLUP of x and the standard deviation of the measurement error, respectively. The precision plots are framed along with 95% confidence intervals. It shows that MADRS is about half as precise as HDRS, even after recalibration. Furthermore, both scales have a higher precision for lower values than higher BLUP of x. The confidence intervals for the recalibrated new method are wider than that of the reference standard method. The lower standard deviation values and narrower confidence intervals collectively indicate the higher precision of the reference standard method.

**Figure 5:**
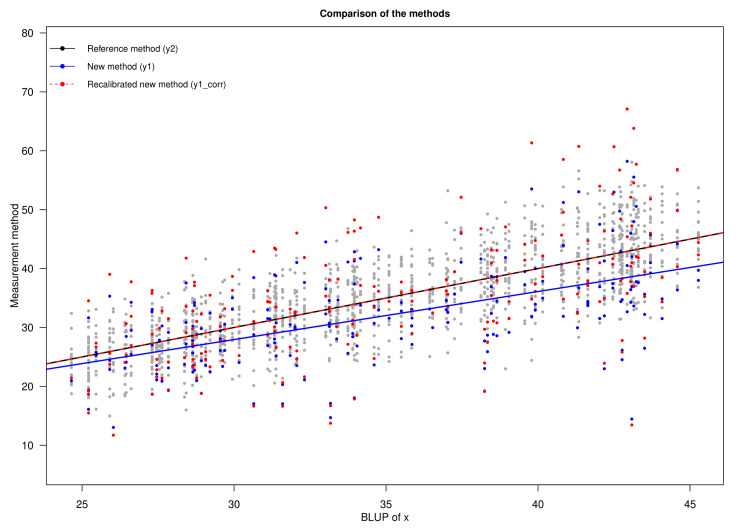
Recalibration of MADRS and its comparison with HDRS The X- and Y-axes represent the BLUP of x and scores assessed with the two measurement methods (i.e., HDRS and MADRS), respectively. The new method (designated as y1) is shown in blue. The reference method (designated as y2) is shown in black. And the recalibrated MADRS (designated as y1_corr) is shown in red. The solid blue, black, and dotted red lines represent the regression lines for the corresponding values. HDRS: Hamilton Depression Rating Scale; MADRS: Montgomery-Åsberg Depression Rating Scale; BLUP: best linear unbiased prediction

Figure 5 compares the methods (i.e., HDRS and MADRS) through a scatter plot after recalibration of the new method (i.e., MADRS). The X- and Y-axes represent the BLUP of x and the values of the different measurement methods, respectively. The scores of the two measurement methods and the recalibrated new method are heteroscedastic. Furthermore, this heteroscedasticity increases with the underlying true latent trait level. The dispersion of HDRS scores is the least among the three methods, and that of the recalibrated MADRS scores is the highest. The regression line of the corrected or recalibrated new method coincides with that of the reference method. It is obvious from the plot that the recalibration of MADRS reduced the bias.

Supplemental materials 1 and 2 display strongly positive correlations between HDRS and MADRS scores at the baseline (r = 0.81, 95% CI = 0.73-0.87, p < 0.001) and 16 weeks (r = 0.91, 95% CI = 0.86-0.94, p < 0.001), respectively. Supplemental material 3 also portrays a strong positive correlation between changes in these scores from baseline (r = 0.72, 95% CI = 0.60-0.80, p < 0.001).

## Discussion

After 16 weeks, all three medicines substantially decreased HDRS and MADRS scores. At both the baseline and last visits, there were significant positive associations between HDRS and MADRS scores. Even the differences across both ratings from the initial assessment were positively correlated. After analyzing the two ratings, our findings mirrored those of Heo et al. [22].

The experimental cohorts received 5-20 mg of vortioxetine and 20-40 mg of vilazodone daily, while the control arm individuals received 10-20 mg of escitalopram. Unlike escitalopram, vilazodone is a partial agonist for the 5-HT1A receptor. Vortioxetine suppresses serotonin transport by interacting with receptors. We discovered that the vortioxetine group had improved depression-related symptoms, overall quality of life, and adherence to therapy in contrast to the other two groups [9,11]. Furthermore, the three medications had similar impacts on several metabolic markers [13].

Regardless of randomization, all trial participants received free medication. This resulted in reduced attrition. All study participants' HDRS and MADRS scores reduced significantly after 16 weeks. During the final visit, the HDRS and MADRS scores exhibited a strong positive correlation (r = 0.91, 95% CI = 0.86-0.94, p < 0.001). The LoA plot revealed a mean score difference of 4.78 (95% CI: 2.61-6.95). This study's interim analysis encountered similar results (mean difference = 5.11, 95% CI: 3.08-7.14) [15]. A meta-analysis by Heo et al. [22] found that HDRS and MADRS are identical in weighing depressive symptoms. Reijnders et al. [23] reported that the HDRS and MADRS can be administered interchangeably to diagnose MDD.

In clinical research, biases can be both differential and proportional. These biases and measurement errors lead to the divergence between observed and predicted values [16]. The LoA plot is developed with the notion that both scales' measurement error variances and precision are identical. It also argues that no proportional bias exists but rather a fixed differential bias [16]. Through the Taffé method, we generated the bias, precision, and comparison plots (shown in Figures 3-5). The computed proportional and differential biases are 0.816 (95% CI: 0.649-0.982) and 3.478 (95% CI: -2.119-9.074), respectively. The heteroscedasticity of HDRS and MADRS scores increases with the underlying true latent trait level. The lower standard deviation values and narrower confidence interval of HDRS collectively indicate its higher precision over MADRS. However, the bias could be eliminated after recalibration of MADRS. Different depression assessment tools have their perks and drawbacks. To select the appropriate scale, one should be well-versed in their respective backgrounds [24]. The Taffé approach aids in contrasting any quantitative scale and visually presents their bias and precision [25].

This study's notable strengths were analyzing HDRS and MADRS scores using Bland and Altman's LoA [14] and the Taffé approach [17]. This is the first inquiry into the bias and precision of these scales. Our study could have been enhanced in a few aspects. First, the open-label trial design may have contributed to dropout rates. Second, we did not assess any depression-specific subjective scales.

## Conclusions

Both HDRS and MADRS scores decreased significantly after 16 weeks of intervention. These scores became more heteroscedastic as the underlying real latent trait level increased. MADRS scores had a higher degree of dispersion. HDRS was more precise than MADRS due to its lower standard deviation and narrower confidence interval. The differential and proportional biases could be reduced with recalibration.

## Figures and Tables

**Figure 6:**
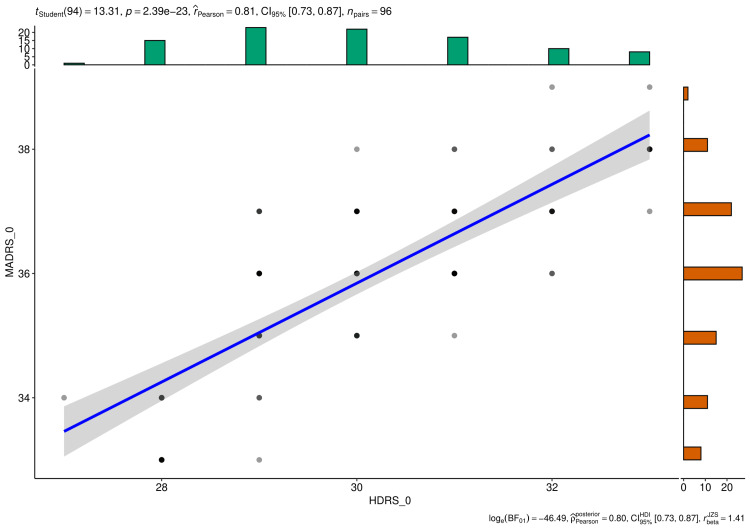
Baseline HDRS and MADRS values of the subjects The scatter plot depicts the association between the baseline HDRS and MADRS scores. The X- and Y-axis histograms demonstrate the number of subjects with baseline HDRS and MADRS scores. Pearson correlation was calculated for the baseline scores for the Hamilton Depression Rating Scale (HDRS_0) and Montgomery-Åsberg Depression Rating Scale (MADRS_0). HDRS: Hamilton Depression Rating Scale; MADRS: Montgomery-Åsberg Depression Rating Scale

**Figure 7:**
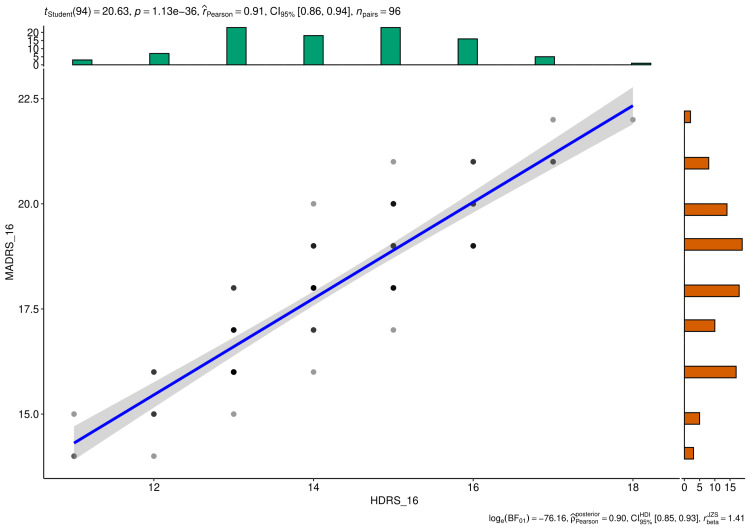
HDRS and MADRS scores at the final visit The scatter plot correlates between the HDRS and MADRS scores at 16 weeks. The histograms on the X- and Y-axis show the number of subjects with their HDRS and MADRS scores at 16 weeks. Pearson correlation was calculated for the 16-week scores for the Hamilton Depression Rating Scale (HDRS_16) and Montgomery-Åsberg Depression Rating Scale (MADRS_16). HDRS: Hamilton Depression Rating Scale; MADRS: Montgomery-Åsberg Depression Rating Scale

**Figure 8:**
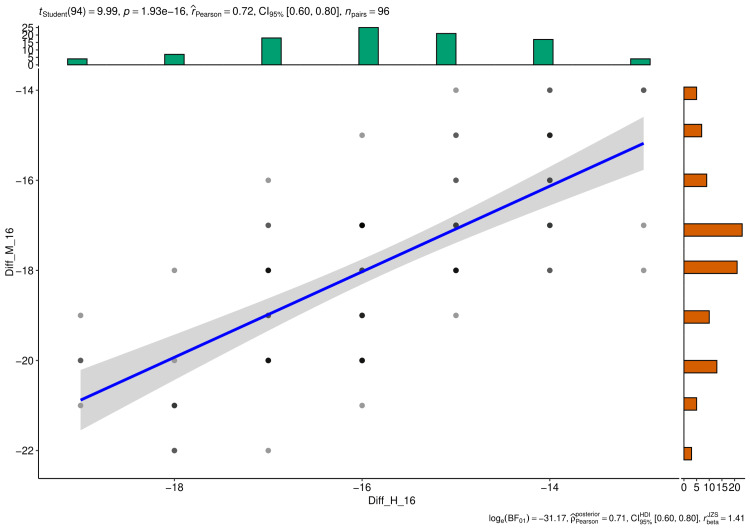
Changes in HDRS and MADRS scores of the study subjects from the baseline The scatter plot depicts the correlation between the study subjects' change in HDRS and MADRS scores from baseline. The X- and Y-axis histograms show the number of participants with changes in HDRS and MADRS scores from baseline at 16 weeks. Pearson correlation was calculated for the change in scores for the Hamilton Depression Rating Scale (Diff_H_16) and the Montgomery-Åsberg Depression Rating Scale (Diff_M_16). HDRS: Hamilton Depression Rating Scale; MADRS: Montgomery-Åsberg Depression Rating Scale

## Appendices

Supplemental material 1

Supplemental material 2

Supplemental material 3

## References

[1] Fries GR, Saldana VA, Finnstein J, Rein T (2023). Molecular pathways of major depressive disorder converge on the synapse. Mol Psychiatry.

[2] Hamilton M (1960). A rating scale for depression. J Neurol Neurosurg Psychiatry.

[3] Montgomery SA, Åsberg M (1979). A new depression scale designed to be sensitive to change. Br J Psychiatry.

[4] Beck AT, Ward CH, Mendelson M, Mock J, Erbaugh J (1961). An inventory for measuring depression. Arch Gen Psychiatry.

[5] Zigmond AS, Snaith RP (1983). The hospital anxiety and depression scale. Acta Psychiatr Scand.

[6] Yesavage JA, Brink TL, Rose TL, Lum O, Huang V, Adey M, Leirer VO (19821983). Development and validation of a geriatric depression screening scale: a preliminary report. J Psychiatr Res.

[7] Martin A, Rief W, Klaiberg A, Braehler E (2006). Validity of the brief patient health questionnaire mood scale (PHQ-9) in the general population. Gen Hosp Psychiatry.

[8] Santi NS, Biswal SB, Naik BN, Sahoo JP, Rath B (2023). An interim analysis of a randomized, open-label study of vilazodone, escitalopram, or vortioxetine for major depressive disorder. Cureus.

[9] Santi NS, Biswal SB, Naik BN, Sahoo JP, Rath B (2024). A randomized controlled trial comparing efficacy and safety of antidepressant monotherapy. Cureus.

[10] Santi NS, Biswal SB, Naik BN, Sahoo JP, Rath B (2023). Quality of life and medication adherence in patients with major depressive disorder: an interim analysis of a randomized study. Cureus.

[11] Santi NS, Biswal SB, Naik BN, Sahoo JP, Rath B (2024). A randomized controlled trial comparing the quality of life and medication adherence in patients on antidepressant monotherapy. Cureus.

[12] Santi NS, Biswal SB, Naik BN, Sahoo JP, Rath B (2023). Metabolic effects of antidepressants: results of a randomized Study’s interim analysis. Cureus.

[13] Santi NS, Biswal SB, Naik BN, Sahoo JP, Rath B (2024). A randomized controlled trial comparing the effects of vilazodone, escitalopram, and vortioxetine monotherapy on the metabolic parameters in patients with major depressive disorder. Cureus.

[14] Bland JM, Altman D (19868). Statistical methods for assessing agreement between two methods of clinical measurement. Lancet.

[15] Santi NS, Biswal SB, Naik BN, Sahoo JP, Rath B (2023). Comparison of Hamilton Depression Rating Scale and Montgomery-Åsberg Depression Rating Scale: baked straight from a randomized study. Cureus.

[16] Taffé P (2021). When can the Bland & Altman limits of agreement method be used and when it should not be used. J Clin Epidemiol.

[17] Taffé P, Halfon P, Halfon M (2020). A new statistical methodology overcame the defects of the Bland-Altman method. J Clin Epidemiol.

[18] Giavarina D (2015). Understanding Bland Altman analysis. Biochem Med (Zagreb).

[19] (2024). R: a language and environment for statistical computing, Vienna, Austria. https://www.r-project.org/.

[20] Lehnert B (2024). Lehnert B. BlandAltmanLeh: plots (slightly extended) Bland-Altman plots. R package version 0.3.1. https://CRAN.R-project.org/package=BlandAltmanLeh.

[21] Taffé P, Peng M, Stagg V, Williamson T (2019). MethodCompare: an R package to assess bias and precision in method comparison studies. Stat Methods Med Res.

[22] Heo M, Murphy CF, Meyers BS (2007). Relationship between the Hamilton Depression Rating Scale and the Montgomery-Åsberg Depression Rating Scale in depressed elderly: a meta-analysis. Am J Geriatr Psychiatry.

[23] Reijnders JS, Lousberg R, Leentjens AF (2010). Assessment of depression in Parkinson's disease: the contribution of somatic symptoms to the clinimetric performance of the Hamilton and Montgomery-Asberg rating scales. J Psychosom Res.

[24] Demyttenaere K, De Fruyt J (2003). Getting what you ask for: on the selectivity of depression rating scales. Psychother Psychosom.

[25] Taffé P (2018). Effective plots to assess bias and precision in method comparison studies. Stat Methods Med Res.

